# ST Depression in the Setting of Subarachnoid Hemorrhage

**DOI:** 10.7759/cureus.19030

**Published:** 2021-10-25

**Authors:** Ala Mustafa, Nathaniel Hitt, Elias Smirlis, Ketan Koranne

**Affiliations:** 1 Internal Medicine, MercyOne North Iowa Medical Center, Mason City, USA; 2 Cardiology, MercyOne North Iowa Medical Center, Mason City, USA

**Keywords:** st depression, subarachnoid hemmorhage, covid 19, 12-lead ecg, angiogram

## Abstract

We present a case report of a patient presenting with subarachnoid hemorrhage whose electrocardiogram (ECG) mimicked non-ST-elevation myocardial infarction. A 36-year-old male with a past medical history of resistant hypertension, previous severe acute respiratory syndrome coronavirus 2 infection, and alcohol abuse presented to the hospital after cardiac arrest. He was taken to the catheterization lab upon arrival and was found to have an unremarkable coronary angiogram. After angiography, computerized tomography (CT) head was performed revealing an acute, large-volume, subarachnoid hemorrhage. Subsequent CT angiogram of the head confirmed this with source noted to be a ruptured aneurysm of the anterior communicating artery. ST depression on ECG has been reported in patients who have suffered a subarachnoid hemorrhage. Although the most common etiology of cardiac arrest is an acute coronary syndrome, other etiologies based on a patient’s past medical history need to remain in the differential. Recognition of ECG changes may lead to earlier diagnosis and decreased mortality in subarachnoid patients.

## Introduction

Misdiagnosis of a subarachnoid hemorrhage may occur more frequently than is realized. Kowalski et al. demonstrated that a subarachnoid hemorrhage was misdiagnosed in 12% of patients [1]. Typically, a patient with suspected subarachnoid hemorrhage will have a computerized tomography (CT) head performed and possibly a lumbar puncture if the CT is negative. In addition, patients suffering from a subarachnoid hemorrhage frequently demonstrate electrocardiogram (ECG) changes [2]. Therefore, it is important to recognize the physical as well as diagnostic findings that may correlate with a subarachnoid hemorrhage. We present here the case of a patient who suffered an out-of-hospital cardiac arrest and was subsequently found to have subarachnoid hemorrhage upon admission to the hospital.

## Case presentation

A 36-year-old male with a past medical history of resistant hypertension, previous severe acute respiratory syndrome coronavirus 2 infection, and alcohol abuse was noted to have "snoring-like" breathing while on the phone with a friend. Emergency medicine services (EMS) were notified and found the patient to be unresponsive. EMS initiated cardiopulmonary resuscitation for a rhythm of pulseless electrical activity. Return of spontaneous circulation was achieved, although the patient continued to intermittently revert to pulseless electrical activity.

**Figure 1:**
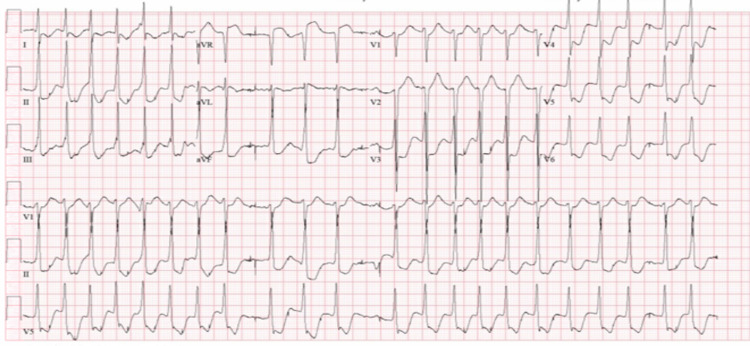
ECG on admission showing diffuse ST segment depressions ECG, electrocardiogram

**Figure 2:**
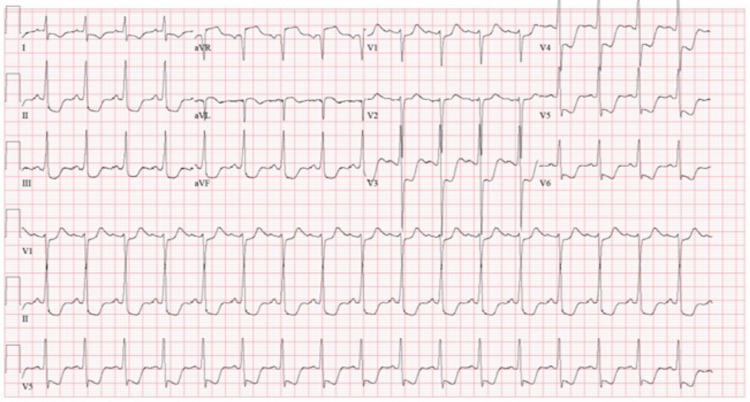
Subsequent ECG demonstrated diffuse ST segment depressions with a mild ST elevation in the aVR lead ECG, electrocardiogram

Prior to arrival to the hospital, EMS staff began compressions and administered two rounds of 1 mg epinephrine and airway was secured with king airway in place. Upon arrival to the hospital emergency department (ED), physical examination demonstrated fixed, dilated pupils, cold extremities, lack of corneal or gag reflex, and a Glasgow Coma Scale of 3. The patient’s rhythm converted to supraventricular tachycardia (SVT) with diffuse ST segment depressions in the ED, as shown in Figure 1. He was electrically cardioverted resulting in sinus tachycardia with a rate of 104 beats per minute. The subsequent ECG demonstrated diffuse ST segment depressions with a mild ST elevation in the aVR lead as shown in Figure 2.

**Figure 3:**
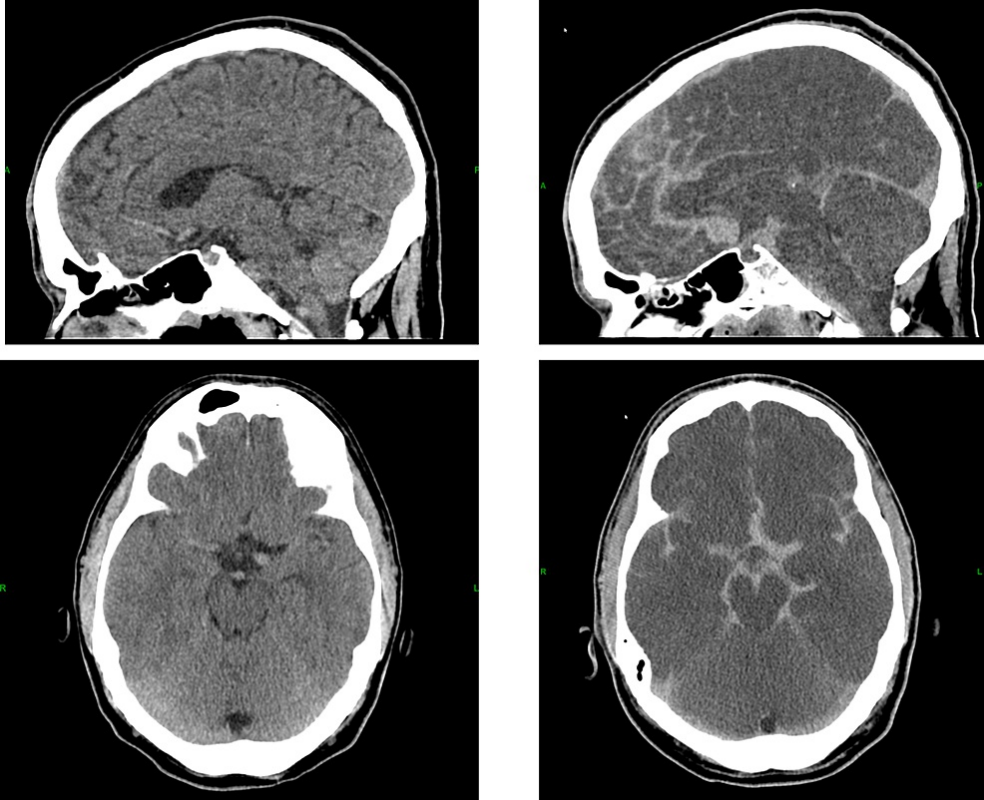
(Right) Shows extensive intracranial subarachnoid hemorrhage visualized during hospitalization, compared to (left) of CT head one year prior CT, computed tomography

After cardioversion, the patient was emergently taken to the cardiac catheterization lab for investigation of his coronary arteries given his ECG findings. Coronary angiography was unremarkable for an etiology of the patient’s cardiac arrest. An ensuing CT of the head was ordered after catheterization and demonstrated a large subarachnoid hemorrhage as seen in Figure 3. A follow-up computerized tomography angiogram (CTA) of the brain was ordered demonstrating extensive intracranial subarachnoid hemorrhage suggesting aneurysmal rupture. The extensive hemorrhage limited assessment for visualization of a small aneurysm although an anterior communicating artery aneurysm was possible. The results of the CTA are shown in Figure 3.

**Figure 4:**
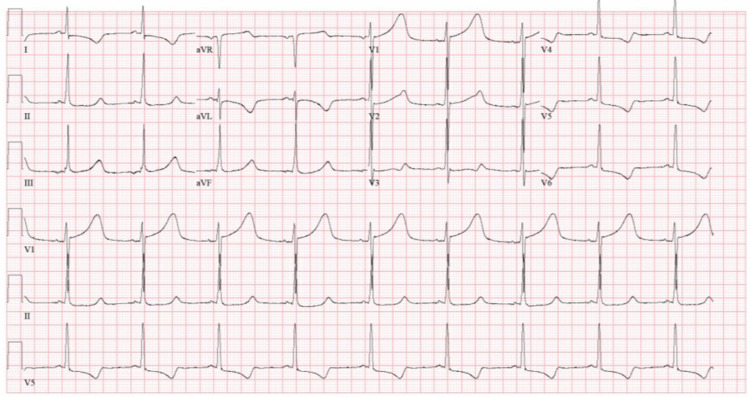
A repeat ECG 27 hours after admission demonstrated a resolution of the ST depressions initially present, QTc prolongation, and T wave inversions in leads I, aVL, and V4-V6 ECG, electrocardiogram

The neurosurgical team was notified of the brain imaging results, but the patient was not a surgical candidate given the extensive nature and that the imaging did not demonstrate cerebral perfusion. A repeat ECG 27 hours after admission demonstrated a resolution of the ST depressions initially present, QTc prolongation, and T wave inversions as demonstrated in Figure 4. Forty-eight hours after admission an electroencephalogram, nuclear medicine brain perfusion scan, and neurologic reflexes confirmed brain death in the patient.

## Discussion

Subarachnoid hemorrhage affects close to 30,000 people in North America yearly. Sixty percent of these patients experience impairment or death. Delays in diagnosis and treatment increase the risk of death by close to 400% [2]. Common symptoms to recognize include "thunderclap" headache, loss of consciousness, meningismus, neck pain, back pain, and retinal hemorrhages [1]. However, cardiac manifestations should not be overlooked. Similar to findings in our case, studies show that 25-90% of patients have ECG abnormalities [2,3]. Our patient's ST depression, mild ST elevation, and T wave inversion represent many of the changes documented in previous subarachnoid hemorrhage cases. Multiple case series document the presence of T wave inversion, ST depression, ST elevation, QT prolongation, U waves, and bundle branch blocks [2-4]. Arrhythmia is seen in up to 63% of subarachnoid patients [2]. Our patient's SVT represents one of the most common types of arrhythmia with atrial fibrillation being most commonly associated with subarachnoid hemorrhage [1]. Tachyarrhythmias have also previously been associated with ischemia, specifically non-ST-elevation myocardial infarction (NSTEMI). The ST elevation in aVR seen in our case represents only 4.59% of subarachnoid patients [2]. In addition to the ECG findings, our patient presented with an initial troponin of 4.5 mcg/L. However, our patient's angiogram was unremarkable. Previous literature has attributed cardiac ischemia to vasospasm, cardiac stunning from catecholamine release, and supply-demand mismatch in tachyarrhythmias [2,5,6]. Cardiac manifestations in general are associated with the severity of subarachnoid hemorrhage, but the presentation of our patient, with NSTEMI and a troponin >1.0 mcg/mL, is associated with a 5 times higher risk of mortality [2]. Early diagnosis is associated with decreased mortality. However, earlier diagnosis in our patient likely would not have changed the outcome based on the time lapse between symptom onset and hospital contact, severity of hemorrhage, initial neurologic exam, and cardiac manifestations. 

## Conclusions

Subarachnoid hemorrhage often goes misdiagnosed. This case highlights that cardiac arrest with ST depression and troponin elevation is not always secondary to coronary artery disease. Cardiac manifestations are an important clue to aid in early diagnosis and prognosis stratification of subarachnoid hemorrhage. ECG changes and arrhythmias can be observed during a patient's initial presentation. Patients like ours, with ST depression and troponin elevation, are at increased risk of death highlighting the importance of early diagnosis of subarachnoid hemorrhage.
